# Community Protections in American Indian and Alaska Native Participatory Research—A Scoping Review

**DOI:** 10.3390/socsci8040127

**Published:** 2019-04-20

**Authors:** Julie A. Beans, Bobby Saunkeah, R. Brian Woodbury, Terry S. Ketchum, Paul G. Spicer, Vanessa Y. Hiratsuka

**Affiliations:** 1 Southcentral Foundation Research Department, 4085 Tudor Centre Dr., Anchorage, AK 99508, USA; (R.B.W.); (V.Y.H.); 2 Chickasaw Nation Department of Health, Research and Public Health Division, 1921 Stonecipher Boulevard, Ada, OK 74820, USA;; 3 Department of Anthropology, University of Oklahoma, 455 West Lindsey, Dale Hall Tower 521, Norman, OK 73019, USA; (T.S.K.); (P.G.S.)

**Keywords:** Indians, North American, Alaska Native, ethics, research, participatory research, tribal sovereignty, scoping review, Indigenous

## Abstract

Experiences with unethical research practices have caused some American Indian and Alaska Native (AIAN) individuals, organizations, and tribes to mistrust health research. To build trust and repair relationships, current research with AIAN peoples often involves participatory research (PR) approaches. This article assesses community-level protections described in the scientific literature on PR involving AIAN communities. A scoping review search in PubMed and PsychInfo for articles published between January 2000 and June 2017 yielded an AIAN PR article dataset. Of 178 articles, a subset of 23 articles that described aspects of community protections were analyzed for descriptions of community-level protection practices. We identified the presence or absence of a description of four community protection measures in each article: a tribal research department, the development of community-level mechanisms for research regulation if not present, community collaboration throughout the research process, and project employment of a community member. The development of community-level mechanisms for research regulation was described in 39% of the articles. Ninety-one percent of these articles described community collaboration during the research process. Seventeen percent included descriptions of all four community-level protection measures. The extent and consistency to which community-level protections are described is variable; the current literature lacks reporting on community-level protection practices specific to tribal communities.

## 1. Introduction

Indigenous populations worldwide have been subjected to research and research practices that fail to address the priorities and health needs of Indigenous communities (Valeggia and Snodgrass 2015; Garrison et al. 2019). Unethical and unregulated research practices with Indigenous populations have led to the development of local, national and international Indigenous-driven frameworks such as the United Nations Declaration on the Rights of Indigenous Peoples that clarify the ethical and legal rights of Indigenous people in research (United Nations General Assembly 2007). Research involving human participants in the United States (US) is guided by federal regulations that require researchers to respect participant autonomy, treat participants fairly, and maximize benefits and minimize harms to participants (Department of Health, Education and Welfare 2014). Although these regulations provide broad protections for individual research participants, they fail to address communityor population-level ethical concerns. When federal regulation standards alone are considered for research protections, leeway exists for research activities that can inadvertently harm communities (Mariella et al. 2009).

Research approaches that promote community participation emerged from the need to address gaps in community-level research protections. A community is more than a group of individuals with shared characteristics: communities also possess an internal structure with identifiable leadership (Ross et al. 2010). Additionally, communities are subject to group-level benefits and harms consequent to the participation of its members in research. In health research for example, if a study design calls for recruitment of individual participants from a community of interest and the research takes place within that specific community, then risks of harm extend beyond the individual to the community as a whole and must be considered. Extending consideration of such harms from the individuals to the communities involved in health research requires the involvement of communities in the research process (Gbadegesin and Wendler 2006).

Participatory research (PR) approaches seek not only to prevent harm, but to actively benefit communities by reimagining the relationship between researchers and participants through the realignment of research goals with community priorities and needs. PR is characterized by a shared commitment to methods that empower and measurably benefit communities through engagement processes, developing capacity, and ceding control of specific research activities to the community (Fisher and Ball 2003; Baum et al. 2006; Israel 2013). Community involvement, a central component of PR, seeks to mitigate inherent power imbalances between researchers and research participants and democratizes decision-making within the research process (Israel et al. 2001; Fisher and Ball 2003; Baum et al. 2006).

### 1.1. Tribal Groups in the US

In the US, tribal groups have several designations that revolve around formal recognition—federal recognition, state recognition and tribes seeking recognition. Federally recognized tribes refer to tribes that have met the US federal regulatory requirements described in 25 C.F.R. §83. These requirements include documentation of the AIAN identity of the group, that the group has a long-standing history as a community, the group has a political structure, and the group has governing documents and unique membership requirements (US Government Publishing O ce 2017). Through federal acknowledgement, the US formally recognizes the tribes right to self-govern, enforce laws, and regulate activities inclusive of health research. In addition to recognizing and promoting an inherent right to self-governance for tribal communities, federally recognized tribes are eligible for services to protect and enhance tribal lands and to improve the well-being of AIAN people as part of the trust responsibility of the US government established in formal government-to-government relations (US Government Publishing Office 2017). Currently, there are 573 federally recognized tribes in the US and over 200 tribes seeking federal recognition (Koenig and Steinberg 2008; US Department of Health and Human Services 2018). Of the over 200 tribes seeking federal recognition, there are over 63 state recognized tribes that have established formal state government relations. State recognition acknowledges the cultural and political history of the tribe and, in some cases, qualifies tribes for federal and state support (Salazar 2016). The formal recognition processes by the US federal and state governments highlight the distinct political designation of AIAN groups in the US and the unique ethical considerations this context holds for health research.

### 1.2. Research in US Tribal Contexts

US federal policy enacted to assimilate AIAN communities and eliminate AIAN cultures has contributed to a legacy of mistrust in federally funded health care for AIAN communities (Hodge 2012; Rhoades and Rhoades 2014; Warne and Frizzell 2014). This mistrust is compounded by the varied experiences of research among tribal communities. For example, in 1979, an AN community sought assistance from researchers in addressing community concerns with alcohol (Foulks 1989). Although the project embraced collaboration between researchers and the community, study findings stigmatizing the AN community were published, without community approval, in a national media outlet (Foulks 1989). In a similar vein, in 1990, Arizona State University researchers were asked by members of an Arizona tribe to investigate the high incidence of diabetes within the tribe (Garrison 2013). Tribal members provided consent and blood samples for diabetes research. Unbeknownst to the participants and community, the samples were also used in controversial studies on the topics of schizophrenia, migration, and consanguinity—unrelated to diabetes (Mello and Wolf 2010; Garrison 2013). These deplorable research practices led not only to stigmatization of the AIAN communities where the research took place, but also to many AIAN people and communities throughout the US contributing to a negative view of health research in tribal communities.

Research in tribal contexts must also consider the sovereign status of tribal governments (Quinn 1990). AIAN tribes maintained their own forms of government prior to European contact and prior to the establishment of the US as a country—many AIAN tribes continue governing their citizens today. The US federal government recognizes the sovereignty of many AIAN tribes, which places AIAN individuals, organizations, and tribes in a unique political position (Quinn 1990). Sovereignty endows tribal governments with inherent authority over a range of research activities involving tribal members, including determination of research objectives and review of manuscripts prior to publication. Thus, researchers and research institutions must approach tribes as governments and recognize the legal force of tribal regulations on research (Fisher and Ball 2005).

Last, the diversity of AIAN communities must be acknowledged. There are 573 federally recognized AIAN tribes and over 63 state-recognized tribes in the US (US Department of Health and Human Services 2018) with distinct languages, cultures, health services infrastructures, public health and health care needs (Goins et al. 2011). Research must be conducted with and for AIAN communities utilizing methods adapted for their specific needs. For AIAN communities, participation in research activities, including the interpretation and dissemination of research findings, acts as a bulwark against misrepresentation and potential stigmatization (Baum et al. 2006). While there are several forms of PR in current practice, this review focuses on two approaches frequently used in research with tribal communities.

### 1.3. Community-Based Participatory Research

Community-based participatory research (CBPR) has been successfully utilized by researchers in partnership with AIAN communities(BlueBirdJernigan2010; Cumminsetal.2010; Burhansstipanov et al. 2013; Jumper-Reeves et al. 2014; Johansson et al. 2015). CBPR aims to benefit communities by aligning research objectives with community needs and by promoting the participation of community members in every stage of the research process (Israel 2013). CBPR is further distinguished by recognition of the community—rather than the individual—as the primary unit of identity, a focus on co-learning among both community members and researchers, and the development of community research capacity (Goins et al. 2011; Israel 2013). Additional guiding principles of CBPR include reliance on an ecological perspective that accounts for locally relevant social determinants of health and a balanced pursuit of research and intervention in order to maximize benefits for all members of the research collaboration (LaVeaux and Christopher 2009; Israel 2013).

### 1.4. Tribal Participatory Research

Although successful projects have been conducted using CBPR approaches in health research with AIAN communities, it has been recognized that CBPR principles alone do not address the needs and political status unique to AIAN communities (LaVeaux and Christopher 2009; Mariella et al. 2009). Tribal Participatory Research (TPR) acknowledges key features specific to AIAN communities which include the history of tribal interactions with the US government and researchers, the sovereign status of tribal governments, and the diversity among AIAN populations (Fisher and Ball 2003). Others have contributed to the development of this research approach specific to AIAN communities (Mariella et al. 2009; Christopher et al. 2011; Claw et al. 2018).

Like CBPR, TPR emphasizes community participation in the research process and the development of community research capacity through training community members in research methods (Fisher and Ball 2003; Mariella et al. 2009; Claw et al. 2018). However, TPR deliberately recognizes the historical and political experiences of AIAN people and communities that contribute to contemporary public health issues. TPR posits that understanding tribal communities’ history and political standing provides a necessary cultural context to the conduct of a research project, interpretation of data, and dissemination of study findings (Fisher and Ball 2003; Christopher et al. 2011; Claw et al. 2018).

Further, TPR explicitly incorporates the recognition of tribal sovereignty by adhering to tribal research oversight processes, including research resolutions, tribal research oversight committees, and the development of research codes (Fisher and Ball 2003; Mariella et al. 2009; Claw et al. 2018). Like CBPR, community participation is promoted in TPR to facilitate equitable power relations between researchers and community members (Fisher and Ball 2003; Christopher et al. 2011). The TPR framework describes the necessity for tribal data governance, oversight of data sharing and dissemination (Mariella et al. 2009). This recognition of tribal sovereignty instills the understanding that AIAN individuals, organizations, and tribes represent not only a distinct culture and background, but also a distinct political designation and standing. Additionally, TPR reminds researchers of the expertise community members have in the cultural and political context relevant to data collected in their communities (Christopher et al. 2011; Claw et al. 2018).

As in CBPR, the dissemination of research findings back to the community is a key aspect; but in recognition of the sovereign status of AIAN tribal groups, TPR explicitly describes the requirement for tribal oversight of publications prior to peer-review journal submission as well as prior review for other modes of dissemination such as abstracts and presentations (Mariella et al. 2009; Claw et al. 2018). Finally, like CBPR, TPR supports the development of community research capacity and emphasizes the use of culturally appropriate measures, interventions, and outcomes (Fisher and Ball 2003; Christopher et al. 2011; Claw et al. 2018).

TPR offers an explicit framework under the umbrella of CBPR for recognizing tribal sovereignty and engaging with the political processes unique to research with AIAN communities. Within the context of the US, AIAN groups are often clumped together as one group despite the cultural and political differences previously outlined. Researchers with limited to no knowledge of AIAN historical and political nuance may be unaware of specific protocols or processes that exist within AIAN communities. This lack of knowledge could pose a risk to the AIAN community in which the research takes place and could extend to other AIAN groups as well. The TPR framework provides detailed guidance in addressing the political and cultural considerations of a government-to-government relationship that could be missed under the guidance of CBPR alone.

### 1.5. Community-Protection Descriptions in PR in AIAN Communities

PR approaches, such as CBPR and TPR, were developed in part as a response to unethical research practices that harmed or failed to benefit communities participating in research. Unsurprisingly then, the literature frequently describes PR in terms of its effectiveness as a strategy for addressing these concerns. Although frameworks and approaches such as CBPR and TPR exist, and researchers broadly report following PR guidelines, providing documentation of explicit examples of how these approaches are applied not only holds the researcher accountable for abiding by PR practices, but models transparent research practices and further empowers AIAN communities that participate in research by explicating their oversight of the research project. This article examines the inclusion of community-level protection descriptions in the scientific literature on participatory health research involving AIAN communities.

## 2. Materials and Methods

### 2.1. Context for Scoping Review on PR with AIAN Communities

The Center for the Ethics of Indigenous Genomic Research (CEIGR) is a Center of Excellence in the Ethical, Legal and Social Implications of Research in the US. The University of Oklahoma collaborated with three AIAN community research groups in the US to form CEIGR. The AIAN community research groups include: Southcentral Foundation in Anchorage, Alaska; the Chickasaw Nation Department of Health in Ada, Oklahoma; and Missouri Breaks Industries Research Incorporated in Eagle Butte, South Dakota. The work of CEIGR is supported by an external advisory committee comprised of researchers and clinicians with experience on research and health care with AIAN populations. As CEIGR and the external advisory committee began dialogue during the initiation of the center, the group became aware of the different forms of PR practiced by the various members of CEIGR. To come to a shared understanding and to review the space where CEIGR work would be placed, the group recognized the need for a review of PR with AIAN communities. This scoping review was conducted by AIAN researchers and AIAN-based research staff. This manuscript was reviewed and approved by Southcentral Foundation and Chickasaw Nation research pre-publication review committees.

### 2.2. Scoping Review

Scoping studies seek to rapidly map key concepts in a research area to identify strengths and gaps in the literature, determine the need for systematic reviews, and inform policy, practice, and/or research (Arksey and O’malley 2005; Levac et al. 2010). This scoping study used a five-stage framework which included: (1) determining the study question and purpose; (2) developing and conducting the search strategy; (3) selecting articles for extraction; (4) charting of data; (5) collating, summarizing, and reporting results (Arksey and O’malley 2005; Levac et al. 2010). Expert consultation was incorporated throughout all five stages of the scoping study.

**Figure 1.**
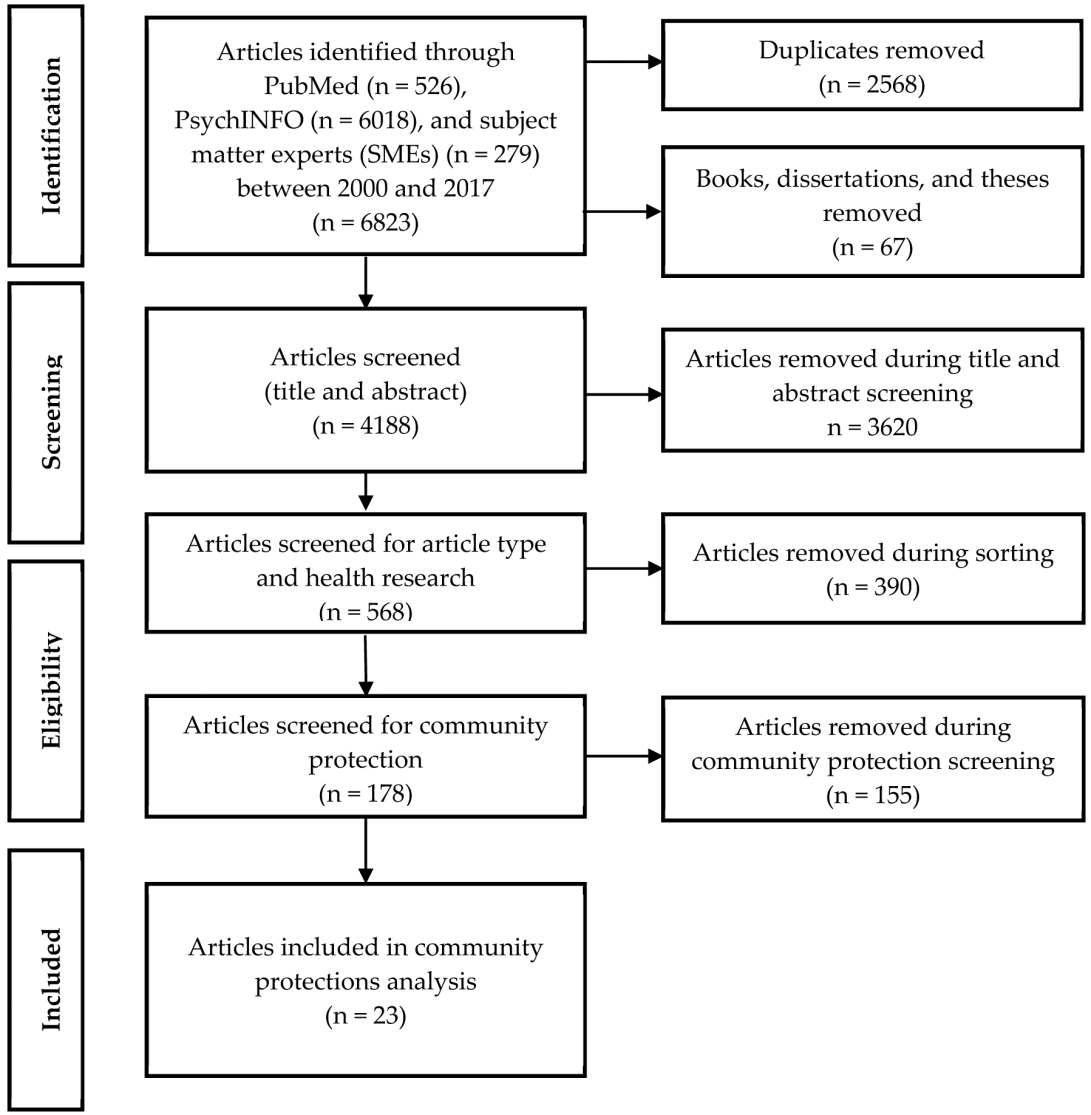
Article selection flow chart.

PubMed and PsychINFO were searched for articles published between 1 January 2000 and 30 June 2017 that contained Medical Subject Headings (MeSH) terms or keywords related to both AIAN populations in the US and PR. Topic-related search terms included: “community based participatory research”, “community engagement”, “participatory action research”, “community institutional relations”, “community participation”, and “action research.” Population-related search terms included: “Alaska Natives”, “Inuits”, and “Indians, North American”, “Indigenous, North American”, “American Indian”, and “Native American.” All fields were searched for these keywords. Results were not limited by document type. Articles and journals were recommended for inclusion by researchers and subject matter experts (SMEs) within CEIGR and the CEIGR external advisory committee which included non-indexed Indigenous journals and articles. These searches, in combination with articles recommended by SMEs, yielded 4188 unique documents (Figure 1). Two researchers (R.B.W. and T.S.K.), with a third as moderator (V.Y.H.) to resolve disputes, conducted article review, selection, and data charting. Articles were reviewed and screened for relevance to topics, approaches, and populations of interest through successive reviews of article title, keywords, and abstract. Next, articles were sorted by type and topic limited to articles describing primary research on health-related topics. During article review, only articles based in the US were included and all others were removed. SMEs were consulted in the development of search strategies, inclusion/exclusion criteria, and the data charting form.

A form was developed to guide the data charting process that included 60 fields, 44 of which related to the principles of CBPR, TPR and other key PR approaches. Fields were marked as either description present or absent. Reviewers consulted one another in cases of uncertainty to account for the variability in how community settings and research processes are described in the literature. If marked present, this indicates the article included a description of the field of interest. If marked absent, this indicated that the article did not include a description of the field of interest. It should be noted that a determination of absence does not mean that the action was not carried out, only that it was not reported in the published article. Once data charting was complete, the dataset was cleaned in preparation for analysis (Figure 1). The resultant dataset included 178 articles that reported on the use *Soc. Sci.* **2019**, *8*, x FOR PEER REVIEW 6 of 18 of PR approaches in primary research on health-related topics involving AIAN people.

### 2.3. Analysis of Ethics of PR in AIAN Communities

Since PR was developed in part to protect the community from research harm, we assessed the inclusion Since PR was developed in part to protect the community from research harm, we assessed the of descriptions of adherence to PR principles in research studies conducted with AIAN inclusion of descriptions of adherence to PR principles in research studies conducted with AIAN communities (Table 1). The dataset of 178 articles for the PR scoping review was screened to include only communities (Table 1). The dataset of 178 articles for the PR scoping review was screened to include those only those that had the presence of all the following selection criteria:

- Community group involved in research project.
- Community-level decision-making power over the research project.
- Ongoing input from community members for project described.
- Tribal-level regulation over research project.
- Relationships established between community and researchers prior to project initiation.
- Relationships between community and researchers maintained beyond project conclusion.

From the 60 fields available in the charted data of the scoping review described above, J.A.B., under the direction of V.Y.H., selected the criteria based on CBPR and TPR principles outlined in Table 1. It is important to note that TPR includes the CBPR principles outlined and also encompasses the additional principles of recognizing tribal sovereignty and the authority this carries in research oversight and data governance, as well as, recognizing the cultural diversity of AIAN people. Table 1 shows the alignment of the selection criteria with specific CBPR and/or TPR principles with an “X”.

We then assessed the 23 articles that met the inclusion criteria of involving the presence of the descriptions of key principles of CBPR and TPR (Table 1). Within the 23 articles, we documented the presence or absence of descriptions of activities related to community protection: (a) tribal research department (b) the development of community-level mechanism for regulating research if not present, (c) community collaboration throughout the research process, and (d) project employment of a community member.

### 2.4. Measures

All articles meeting the inclusion criteria were analyzed for further descriptions of community-level involvement. *Research Approach* was categorized as utilizing PR, CBPR, and/or TPR (Table 2). We categorized the *Research Setting* as either rural or urban. Rural included settings describing reservations, and/or villages in Alaska and rural tribal communities to be inclusive of state-recognized tribes that do not have reservations. The presence of *Tribal Research Department* was used to indicate a degree of familiarity with the research process. Yes (Y) was marked if the article explicitly reported that the participating community had a tribal research department *or* if information presented in the article strongly suggested that the participating community had a tribal research department. No (N) was marked if the article explicitly reported that the participating community did not have a tribal research department *or* if information presented in the article strongly suggested that the participating community did not have a tribal research department. Not Reported (NR) was recorded if the article did not report whether the community had a tribal research department *and* if the article did not present information that strongly suggested that the participating community had or did not have a tribal research department.

For the column, *Development of community-level mechanism for regulating research if not present* (Table 2), a “Y” response referred to articles that explicitly stated research partners assisted in the development of a tribal mechanism for regulating research *or* if information presented in the article strongly suggested that the research partners engaged in this activity. An “N” referred to articles explicitly stating that research partners did not assist in the development of a tribal mechanism for regulating research *or* if information presented in the article strongly suggested that the research partners did not engage in this activity. Responses of “NR” were entered if the article did not report whether research partners assisted in the development of tribal mechanisms for regulating research *and* if the article did not present information that strongly suggested that the research partners engaged or did not engage in this activity. Examples of tribal mechanisms for regulating research included tribal institutional review boards (IRBs), tribal research codes, tribal resolutions, formal contracts with tribal governments, research agreements, data sharing agreements, and memoranda of agreement/understanding.

*Community collaboration throughout the research process* (Table 2) is the third column header. An entry of “Y” was made if the article explicitly stated that the research project involved a community member who functioned as a tribal liaison/facilitator tasked with responsibilities including, but not limited to, promoting community engagement in the research process and communication between researchers and the community *or* if information provided in the article strongly suggested that a community member filled this role. Entries of “N” indicate whether the article explicitly stated that the research project involved a community member who functioned as a tribal liaison/facilitator tasked with responsibilities including, but not limited to, promoting community engagement in the research process and communication between researchers and the community *or* whether information provided in the article strongly suggested that no community member filled this role. An “NR” was entered if the article did not report whether the researchers employed a community member to function as a tribal liaison/facilitator *and* if no information provided in the article strongly suggested that a community member filled or did not fill this role.

The column, *Project employment of a community member* (Table 2), has entries of “Y” if the article explicitly stated that community members were involved in the selection or development of study design, methods, or approach *or* if information presented in the article strongly suggested that community members were involved in these activities. An “N” was entered if the article explicitly stated that community members were not were involved in the selection or development of study design, methods, or approach *or* if information presented in the article strongly suggested that community members were not involved in these activities. Entries of “NR” were used if the article did not report whether community members were involved in the selection or development of study design, methods, or approach *and* if the article did not present information that strongly suggested that community members were or were not involved in these activities.

## 3. Results

Of the 178 articles in the original scoping review on PR in AIAN communities, 23 articles included a description of:

- Community group involved in research project;
- Community-level decision-making power over the research project;
- Ongoing input from community members for project described;
- Tribal-level regulation over research project;
- Relationships established between community and researchers prior to project initiation, and
- Relationships between community and researchers maintained beyond project conclusion.

Publication dates for the 23 articles ranged from 2000 to 2016. Study characteristics of community protections in PR are in Table 2.

Use of a CBPR research framework was reported in 87% (20/23) of the articles. Of the 20 articles that used CBPR, 4 articles reported utilizing a TPR or TPR-like framework in addition to CBPR framework. The use of a general PR approach was reported in 13% (3/23) of the articles. Of the 21 articles published in 2005 or later, 20 reported using a CBPR framework.

Seventy-four percent (17/23) of articles described studies or projects occurring in communities located in rural settings, and 26% (6/23) described research occurring in a combination of rural and urban settings. No articles described research in an urban setting only.

Twenty-six percent (6/23) of articles reported that the community involved in the research project had a tribal research department, 9% (2/23) reported not having a research department, and 65% (15/23) did not include descriptions of a tribal research department. Thirty-nine percent (9/23) of articles reported the development of community-level mechanisms for regulating research if not present, 4% (1/23) reported that a community-level mechanism for regulating research was not developed if it did not exist, and 57% (13/23) did not report on the development of a community-level mechanism for regulating research if not present.

Ninety-one percent (21/23) reported that the community members collaborated with researchers throughout the research process. In the other 9% (2/23) of articles, this information was not reported. Fifty-seven percent (13/23) of the articles reported project employment of a community member. Four percent (1/23) reported not employing a community member, and 39% (9/23) of articles did not report employing a community member.

One article did not include descriptions of any of the four community-level protection measures and seventeen percent of articles (4/23) included descriptions of all four community-level protection measures.

## 4. Discussion

Scoping reviews can be used to identify gaps in the existing literature (Arksey and O’malley 2005). This review highlights the variability on the reporting of health-related PR that involves AIAN communities and underscores an absence of reporting on research projects following principles related to PR. It is notable that of the 178 articles included in the original scoping review on PR in AIAN communities, only 23 articles or 13% met the community-protection description inclusion criteria for this analysis. Within CBPR and other PR approaches, there appears to be a lack of attention to reporting community-level protection processes and practices and this review suggests a need for improved reporting guidelines.

Communicating engagement practices when reporting on study findings in health research with AIAN communities is necessary to provide a framework for researchers unfamiliar with oversight processes unique to AIAN communities, to empower AIAN communities that participate in health research, and hold researchers working with AIAN communities accountable to PR principles developed to protect communities from research harms. Researchers unfamiliar with engaging with AIAN communities may not realize how impactful the socio-political experiences with research can be in shaping collaborations and participatory projects. Reporting explicit practices can provide a model for others to consider when engaging AIAN communities in research.

To account for the time and effort in developing research oversight processes and practices deemed acceptable by AIAN communities and for researchers to carry out mutually beneficial health research, it is vital that resultant processes and practices are reported in the academic literature. Reporting these processes and practices does not only promote the transparency necessary for conducting research in AIAN communities (Fisher and Ball 2003; Christopher et al. 2011; Claw et al. 2018), but honors the effort the community put forth in the development and conduct of the research project.

Further, describing these efforts in academic literature allows for AIAN community preferences to have impact beyond the research project. Many AIAN teachings are upheld through oral stories that encompass important life lessons, which are passed on throughout generations. To mirror this concept, researchers have the ethical obligation to uphold their responsibility of passing on the important lessons learned from working with an AIAN community for the benefit of other AIAN communities and researchers.

Last, the lack of reporting of community protection efforts can impact research practices with other Indigenous peoples and groups other than AIAN communities. Due to the unique political designation of AIAN peoples in the US, AIAN tribal groups have a designated structure within their communities that allow for formal processes to be developed to govern research and the sovereignty to enforce these processes. However, other groups may not have the structure in place to develop such processes and may not have the legal jurisdiction to enforce research oversight processes.

### 4.1. Research with Urban AIAN Communities

Ensuring research involving AIAN people located in urban settings utilizes appropriate community-level protections can present distinct challenges. Of the 23 articles included in this analysis, none involved an urban group alone; and six included urban groups in addition to rural groups (Orians et al. 2004; Horn et al. 2008; Daley et al. 2010; Fleischhacker et al. 2011; Fleischhacker et al. 2012; Griese et al. 2016). Urban AIAN groups are often dispersed throughout an urban center, making it diffcult to identify and partner with urban AIAN groups (Castor et al. 2006). Despite this difficulty, inclusion of AIAN groups in urban areas is important because a large number of AIAN people reside in an urban setting (Urban Indian Health Commission 2007). AIAN people living in urban areas may reside and gather in several locations. If studies are to recruit truly representative samples from these groups, more time may be required to build relationships across the many organizations that serve AIAN people living in urban areas.

Moreover, AIAN people living in urban settings may represent several tribes with different political structures and cultural norms. As a result, it can be challenging to determine which entities have the sovereign right and responsibility of providing appropriate research protections for these groups and to deploy protections that account for the political and cultural differences between groups. It is also unclear whether tribes have a responsibility in protecting their individual tribal members who reside in urban centers. Further, researchers may have a responsibility to seek approval from tribal leadership of AIAN tribal members residing in urban settings.

### 4.2. Community-Level Research Review

Community-level research review has been used as a means to mitigate tribal community-level concerns throughout the research process (Hiratsuka et al. 2017). While community-level research review has appeared in PR, the time-intensive nature of this process is sometimes described by researchers as a barrier and may inhibit its widespread uptake (Wolf et al. 2005). Although consensus-building activities and long-term partnerships take time, they have important benefits for communities and are key elements in PR (Bromley et al. 2015). Further, investigators may not be able to accurately assess the degree of risk and benefit for the community for proposed research that is provided by a community-level research review (Gbadegesin and Wendler 2006). Reporting on abiding by this PR principle holds the researcher accountable and shows respect for tribal oversight.

Nine of the articles in this analysis included descriptions of a system or procedure for regulating research and a tribal IRB or tribal community-level review was not available (Table 2). Tribal IRBs or a tribal community-level review of research can provide an expansion on the Common Rule that includes requirements for review and approval of all publications and presentations resulting from the research (Chadwick et al. 2014; Angal et al. 2016) A tribal community-level review in health research is an acknowledgement of an AIAN groups’ sovereign right to oversee research to protect tribal citizens from individual and/or group harm, (Hull and Wilson 2017) and provides a way for AIAN communities to stay informed of research results and dissemination activities (Angal et al. 2016). It should be noted that five of the six articles including an urban sample did not provide a description of a system or procedure for regulating research and a tribal IRB or tribal community-level research review was either not available or not reported as being sought (Orians et al. 2004; Legaspi and Orr 2007; Fleischhacker et al. 2011; Fleischhacker et al. 2012).

Tribal IRBs, other forms of research review committees, and written policies to oversee research activities are approaches that some tribal communities are adopting to exert sovereign authority over research (Chadwick et al. 2014). These AIAN-governed bodies may be charged not only with protecting individual research participants but also the tribal community as a whole, ensuring that potential benefits are not negated by inappropriately conducted research (Morton et al. 2013). Written policies provide a mechanism to address federal funding agency requirements such as broad data sharing, data ownership, and newer requirements such as single IRB review for multi-site research studies (Wolinetz and Collins 2017) which may conflict with tribal protocols and process, or even tribal protections completely (Hull and Wilson 2017).

### 4.3. Community–Academic Partnerships in Research

Both CBPR (Israel 2013) and TPR (Fisher and Ball 2003; Mariella et al. 2009) describe community–academic partnerships as essential aspects of PR. Twenty-one of the 23 articles in this analysis includes a description of community member collaboration throughout the research process, reinforcing the notion that effective partnerships promote successful community-driven projects. These community–academic partnerships underscore the establishment of trust after many AIAN communities have declined to participate in research studies due to past harms (Garrison and Cho 2013; James et al. 2014; Blacksher et al. 2016; Morales et al. 2016; Brockie et al. 2017).

Community–academic partnerships have important points to consider that may be unfamiliar to some. For example, developing the relationships crucial to the success of a community–academic partnership requires an extended planning period and cost commitments (Chadwick et al. 2014; Bromley et al. 2015). The recruitment and training efforts necessary for developing both community research capacity and researcher knowledge of community culture are similarly time-intensive and incur additional cost (Thomas et al. 2011). Tribal review of research proposals and products require planning around set meeting dates that may extend timelines (LaVeaux and Christopher 2009). Increased time commitments and costs are among the most commonly cited learning curves necessary to build mutually beneficial community–academic partnerships in research (Drahota et al. 2016).

Interestingly, several articles did not report on project employment of a community member despite most articles reporting community collaboration. Most past research failed to include AIAN communities as equal partners, and rarely did an AIAN community have the capacity to take the lead on health research projects (Blue Bird Jernigan et al. 2015). Employing a community member as part of the research project can help to shift this narrative. Additionally, a community member employed by the research project may be able to navigate previously reported challenges encountered when conducting research with community–academic partnerships such as the absence of shared goals and expectations, a lack of clarity regarding individual roles and responsibilities, and other consequences of poor communication (Drahota et al. 2016). Finally, the sharing of knowledge between community members and researchers further establishes trust by providing bidirectional learning and understanding of cultural differences through the PR approach.

### 4.4. Limitations and Future Research

This review utilized a dataset from articles identified in a study on the scope of literature on PR practices with AIAN people in the US and may not represent all literature on PR with AIAN people. Moreover, articles identified and included in the main dataset did not focus specifically on the ethical aspects of PR. Future reviews on PR practices may benefit by including non-indexed databases, more inclusive search terms, utilize an extended publication time period, include Indigenous groups globally and expand the search of practices beyond US based articles. It is important to note that the same research project or group of related projects may be described by more than one article in this review.

Articles included in this analysis were selected based on the presence of inclusion criteria described previously, which may not reflect the actual practice that took place but highlights what is and what is not reported in the literature. Further, this study found variation in the reporting practices on community protection efforts used in health research with AIAN communities which may call to question the need for overall reporting guidelines. Further inquiry on reporting practices in general may be warranted.

## 5. Conclusions

This review reveals the inadequate reporting of PR practices in the literature on health research with AIAN communities. Several authors have proposed that PR methods such as CBPR and TPR are appropriate mechanisms to protect AIAN communities engaged in research (Pacheco et al. 2013; Simonds and Christopher 2013); however, the requirement to report on how PR approaches were enacted is missing. It is vital to include explicit descriptions of community protection practices in reporting on PR with AIAN communities to uphold the necessity for mutually beneficial research and inclusion of the community voice throughout the research project outlined by PR approaches. This perspective is particularly applicable to AIAN communities, many of which are sovereign nations with an inherent right to self-determination, including determination of how and what kind of research will be conducted in their communities. Standards of research practice reporting to include community protections in research involving Indigenous populations is warranted to document and evaluate the adherence to best practices and ethical frameworks aimed at meaningful and ethical engagement with Indigenous people.

## Figures and Tables

**Table 1.** Inclusion criteria alignment with participatory research (PR) principles.

PR Principles	Community Group Involved in Research Project	Community-Level Decision-Making Power over the Research Project	Ongoing Input from Community Members for Project	Tribal-Level Regulation over Research	Relationships Established Prior to Project	Relationships Maintained beyond Described Project
**Principles of community-based participatory research (CBPR)** (Israel 2013)						
Recognize community as a unit of identity	**X**					
Build on community strengths and resources	**X**					
Equitable partnership in all research phases and involves power-sharing process		**X**	**X**			
Promotes co-learning and capacity building among all partners	**X**		**X**			
Integrates and achieves a balance between research and action for the mutual benefit of all partners	**X**	**X**	**X**			
Emphasizes health problems of local relevance that attend to multiple determinants of health and disease	**X**	**X**	**X**		**X**	
Involves systems development through a cyclical and iterative process	**X**	**X**	**X**			
Disseminates findings and knowledge gained to all partners and involves all partners in the dissemination process		**X**	**X**			**X**
Requires a long-term process and commitment to sustainability.						**X**
Addresses issues of race, ethnicity, racism, and social class and embraces “Cultural Humility”	**X**			**X**		
**Tribal Participatory Research (TPR) Principles that Supplement CBPR** (Fisher and Ball 2003; Mariella et al. 2009; Christopher et al. 2011; Claw et al. 2018)						
Recognition of tribal sovereignty (i.e., research oversight, data governance)		**X**		**X**	**X**	
Understand implications of diversity of AIAN people	**X**	**X**	**X**		**X**	

**Table 2.** Community-level protection measures across scoping review articles (N = 23).

Article	Research Approach	Setting	Tribal Research Department	Development of Community-Level Mechanism for Regulating Research If Not Present	Community Collaboration throughout the Research Process	Project Employment of a Community Member
(Quigley et al. 2000)	PR	Rural	NR	Y	Y	NR
(Orians et al. 2004)	PR	Rural and Urban	NR	N	Y	Y
(Schell et al. 2005)	CBPR and PR	Rural	Y	Y	Y	Y
(Schell et al. 2007)	CBPR	Rural	Y	Y	Y	Y
(Legaspi and Orr 2007)	CBPR and TPR	Rural	Y	NR	NR	Y
(Christopher et al. 2008)	CBPR	Rural	NR	NR	NR	NR
(Horn et al. 2008)	CBPR	Rural and Urban	N	Y	Y	Y
(Schroepfer et al. 2009)	CBPR	Rural	NR	NR	Y	NR
(Matloub et al. 2009)	CBPR	Rural	NR	NR	Y	NR
(Thomas et al. 2009)	CBPR and TPR	Rural	NR	Y	Y	Y
(Perry and Hoffman 2010)	CPBR	Rural	NR	Y	Y	Y
(Brown et al. 2010)	CBPR	Rural	NR	NR	Y	NR
(Daley et al. 2010)	CBPR	Rural and Urban	NR	NR	Y	N
(Fleischhacker et al. 2011)	CBPR	Rural and Urban	N	NR	Y	Y
(Walters et al. 2012)	CBPR	Rural	Y	Y	Y	NR
(Rink et al. 2012)	CBPR	Rural	NR	NR	Y	NR
(Fleischhacker et al. 2012)	CBPR	Rural and Urban	NR	NR	Y	Y
(Tingey et al. 2014)	CBPR	Rural	Y	NR	Y	NR
(Ravenscroft et al. 2015)	CBPR	Rural	NR	Y	Y	Y
(Cordova et al. 2015)	CBPR	Rural	NR	NR	Y	Y
(Donovan et al. 2015)	CBPR and TPR	Rural	NR	Y	Y	Y
(Griese et al. 2016)	CBPR and TPR	Rural and Urban	Y	NR	Y	Y
(Kelley et al. 2016)	PR	Rural	NR	NR	Y	NR

NR = not reported, Y = yes, N = no.

## References

[R1] Angal Jyoti, Petersen Julie M., Tobacco Deborah, Elliott Amy J., and Network Pass. 2016 Ethics Review for a Multi-Site Project Involving Tribal Nations in the Northern Plains. Journal of Empirical Research on Human Research Ethics: JERHRE 11: 91–96. 26928897 10.1177/1556264616631657 PMC4917412

[R2] Arksey Hilary, and O’malley Lisa. 2005 Scoping studies: Towards a methodological framework. International Journal of Social Research Methodology 8: 19–32.

[R3] Baum Fran, Macdougall Colin, and Smith Danielle. 2006 Participatory action research. Journal of Epidemiology and Community Health 60: 854–57. 16973531 10.1136/jech.2004.028662 PMC2566051

[R4] Blacksher Erika, Nelson Charlene, Van Dyke Emily, Echo-Hawk Abigail, Bassett Deborah, and Buchwald Dedra. 2016 Conversations about Community-Based Participatory Research and Trust: “We Are Explorers Together”. Progress in Community Health Partnerships: Research, Education, and Action 10: 305–9. 10.1353/cpr.2016.0039 27346777

[R5] Blue Bird Jernigan Valarie. 2010 Community-Based Participatory Research with Native American Communities: The Chronic Disease Self-Management Program. Health Promotion Practice 11: 888–99. 19376928 10.1177/1524839909333374 PMC4718735

[R6] Blue Bird Jernigan Valarie, Peercy Michael, Branam Dannielle, Saunkeah Bobby, Wharton David, Winkleby Marilyn, Lowe John, Salvatore Alicia L., Dickerson Daniel, Belcourt Annie, and 2015 Beyond health equity: Achieving wellness within American Indian and Alaska Native communities. American Journal of Public Health 105 S3: S376–79. 25905823 10.2105/AJPH.2014.302447 PMC4455506

[R7] Brockie Teresa N., Dana-Sacco Gail, Miriam Magaña Lopez, and Wetsit Lawrence. 2017 Essentials of Research Engagement with Native American Tribes: Data Collection Reflections of a Tribal Research Team. Progress in Community Health Partnerships: Research, Education, and Action 11: 301–7. 10.1353/cpr.2017.0035 29056622

[R8] Bromley Elizabeth, Mikesell Lisa, Jones Felica, and Khodyakov Dmitry. 2015 From subject to participant: Ethics and the evolving role of community in health research. American Journal of Public Health 105: 900–8. 25790380 10.2105/AJPH.2014.302403 PMC4386538

[R9] Brown Blakely D., Harris Kari Jo, Harris Jeri Lyn, Parker Martin, Ricci Christiana, and Noonan Curtis. 2010 Translating the Diabetes Prevention Program for Northern Plains Indian Youth through Community-Based Participatory Research Methods. The Diabetes Educator 36: 924–35. 20944056 10.1177/0145721710382582 PMC3285486

[R10] Burhansstipanov Linda, Christopher Suzanne, and Schumacher Ann Sr. 2013 Lessons learned from community-based participatory research in Indian country. Cancer Control 12: 70–76. 10.1177/1073274805012004s10 PMC3544402 16327753

[R11] Castor Mei L., Smyser Michael S., Taualii Maile M., Park Alice N., Lawson Shelley A., and Forquera Ralpha A.. 2006 A nationwide population-based study identifying health disparities between American Indians/Alaska Natives and the general populations living in select urban counties. American Journal of Public Health 96: 1478–84. 16571711 10.2105/AJPH.2004.053942 PMC1522100

[R12] Chadwick Jennifer Q., Copeland Kenneth C., Daniel Mary R., Erb-Alvarez Julie A., Felton Beverly A., Khan Sohail I., Saunkeah Bobby R., Wharton David F., and Payan Marisa L.. 2014 Partnering in Research: A National Research Trial Exemplifying Effective Collaboration with American Indian Nations and the Indian Health Service. American Journal of Epidemiology 180: 1202–7. 25389367 10.1093/aje/kwu246 PMC4262439

[R13] Christopher Suzanne, Gidley Allison L., Letiecq Bethany, Smith Adina, and Mccormick Alma Knows. 2008 A cervical cancer community-based participatory research project in a Native American community. Health Education & Behavior: The Offcial Publication of the Society for Public Health Education 35: 821–34. 10.1177/1090198107309457 18077653

[R14] Christopher Suzanne, Saha Robin, Lachapelle Paul, Jennings Derek, Colclough Yoshiko, Cooper Clarice, Cummins Crescentia, Eggers Margaret J., FourStar Kris, Harris Kari, and 2011 Applying indigenous community-based participatory research principles to partnership development in health disparities research. Family & Community Health 34: 246–55. 21633218 10.1097/FCH.0b013e318219606f PMC5443618

[R15] Claw Katrina G., Anderson Matthew Z., Begay Rene L., Tsosie Krystal S., Fox Keolu, and Garrison Nanibaa’ A.. 2018 A framework for enhancing ethical genomic research with Indigenous communities. Nature Communications 9: 2957. 10.1038/s41467-018-05188-3 PMC6063854 30054469

[R16] Cordova Felina M., Joshweseoma Lori, Teufel-Shone Nicolette I., and Coe Kathryn. 2015 Using a Community-Based Participatory Research Approach to Collect Hopi Breast Cancer Survivors’ Stories. American Indian Culture and Research Journal 39: 97–109.

[R17] Cummins Crescentia, Doyle John, Kindness Larry, Lefthand Myra J., Bear Dont Walk Urban J., Bends Ada L., Broadaway Susan C., Camper Anne K., Fitch Roberta, Ford Timothy E., and 2010 Community-based participatory research in Indian country: Improving health through water quality research and awareness. Family & Community Health 33: 166–74. 20531097 10.1097/FCH.0b013e3181e4bcd8 PMC3070444

[R18] Daley Christine Makosky, James Aimee S., Ulrey Ezekiel, Joseph Stephanie, Talawyma Angelia, Choi Won S., Greiner Allen K., and Coe Kathryn M.. 2010 Using Focus Groups in Community-Based Participatory Research: Challenges and Resolutions. Qualitative Health Research 20: 697–706. 20154299 10.1177/1049732310361468 PMC2947156

[R19] Department of Health, Education and Welfare. 2014 The Belmont Report. Ethical principles and guidelines for the protection of human subjects of research. The Journal of the American College of Dentists 81: 4–13. 25951677

[R20] Donovan Dennis M., Thomas Lisa Rey, Sigo Robin Little Wing, Price Laura, Lonczak Heather, Lawrence Nigel, Ahvakana Katie, Austin Lisette, Lawrence Albie, Price Joseph, and 2015 Healing of the canoe: Preliminary results of a culturally grounded intervention to prevent substance abuse and promote tribal identity for Native youth in two pacific northwest tribes. American Indian and Alaska Native Mental Health Research (Online) 22: 42–76. 25768390 10.5820/aian.2201.2015.42 PMC4374439

[R21] Drahota Amy, Meza Rosemary D., Brikho Brigitte, Naaf Meghan, Estabillo Jasper A., Gomez Emily D., Vejnoska Sarah F., Dufek Sarah, Stahmer Aubyn C., and Aarons Gregory A.. 2016 Community-Academic Partnerships: A Systematic Review of the State of the Literature and Recommendations for Future Research. The Milbank Quarterly 94: 163–214. 26994713 10.1111/1468-0009.12184 PMC4941973

[R22] Fisher Philip A., and Ball Thomas J.. 2003 Tribal participatory research: Mechanisms of a collaborative model. American Journal of Community Psychology 32: 207–16. 14703257 10.1023/b:ajcp.0000004742.39858.c5

[R23] Fisher Philip A., and Ball Thomas J.. 2005 Balancing empiricism and local cultural knowledge in the design of prevention research. Journal of Urban Health: Bulletin of the New York Academy of Medicine 82 S3: iii44–iii55. 15933330 10.1093/jurban/jti063 PMC3455902

[R24] Fleischhacker Sheila, Vu Maihan, Ries Amy, and Mcphail Ashley. 2011 Engaging tribal leaders in an American Indian healthy eating project through modified talking circles. Family & Community Health 34: 202–10. 21633212 10.1097/FCH.0b013e31821960bb

[R25] Fleischhacker Sheila, Byrd Randi R., Ramachandran Gowri, Vu Maihan, Ries Amy, Bell Ronny A., and Evenson Kelly R.. 2012 Tools for Healthy Tribes: Improving Access to Healthy Foods in Indian Country. American Journal of Preventive Medicine 43 S2: S123–29. 22898161 10.1016/j.amepre.2012.05.015 PMC3431552

[R26] Foulks Edward F. 1989 Misalliances in the Barrow Alcohol Study. American Indian and Alaska Native Mental Health Research (Online) 2: 7–17. 10.5820/aian.0203.1989.7 2490286

[R27] Garrison Nanibaa’ A. 2013 Genomic Justice for Native Americans: Impact of the Havasupai Case on Genetic Research. Science, Technology & Human Values 38: 201–23. 10.1177/0162243912470009 PMC5310710 28216801

[R28] Garrison Nanibaa’ A., and Cho Mildred K.. 2013 Awareness and Acceptable Practices: IRB and Researcher Reflections on the Havasupai Lawsuit. AJOB Primary Research 4: 55–63. 24089655 10.1080/21507716.2013.770104 PMC3786163

[R29] Garrison Nanibaa’ A., Māui Hudson, Ballantyne Leah L., Garba Ibrahim, Martinez Andrew, Taualii Maile, Arbour Laura, Caron Nadine R., and Carroll Rainie Stephanie. 2019 Genomic Research through an Indigenous Lens: Understanding the Expectations. Annual Review of Genomics and Human Genetics 20. 10.1146/annurev-genom-083118-015434 30892943

[R30] Gbadegesin Segun, and Wendler David. 2006 Protecting Communities in Health Research from Exploitation. Bioethics 20: 248–53. 17100008 10.1111/j.1467-8519.2006.00501.x

[R31] Turner Goins, R., Garroutte Eva Marie, Fox Susan Leading, Dee Geiger Sarah, and Manson Spero M.. 2011 Theory and Practice in Participatory Research: Lessons from the Native Elder Care Study. The Gerontologist 51: 285–94. 21292753 10.1093/geront/gnq130 PMC3095653

[R32] Griese Emily R., Baete Kenyon Denyelle, and Mcmahon Tracey R.. 2016 Identifying sexual health protective factors among norther plains American Indian youth: An ecological approach utilizing multiple perspectives. American Indian and Alaska Native Mental Health Research (Online) 23: 16–43. 27536896 10.5820/aian.2304.2016.16 PMC5463740

[R33] Hiratsuka Vanessa Y., Beans Julie A., Robinson Renee F., Shaw Jennifer L., Sylvester Ileen, and Dillard Denise A.. 2017 Self-Determination in Health Research: An Alaska Native Example of Tribal Ownership and Research Regulation. International Journal of Environmental Research and Public Health 14: 1324. 10.3390/ijerph14111324 PMC5707963 29088111

[R34] Hodge Felicia Schanche. 2012 No Meaningful Apology for American Indian Unethical Research Abuses. Ethics & Behavior 22: 431–44.

[R35] Horn Kimberly, Mccracken Lyn, Dino Geri, and Brayboy Missy. 2008 Applying community-based participatory research principles to the development of a smoking-cessation program for American Indian teens: “Telling our story”. Health Education & Behavior: The Offcial Publication of the Society for Public Health Education 35: 44–69. 10.1177/1090198105285372 PMC7351117 16740518

[R36] Hull Sara Chandros, and Wilson David R.. 2017 Beyond Belmont: Ensuring Respect for AI/AN Communities Through Tribal IRBs, Laws, and Policies. The American Journal of Bioethics 17: 60–62. 10.1080/15265161.2017.1328531 PMC6097707 28661757

[R37] Israel Barbara A. 2013 Methods for Community-Based Participatory Research for Health. San Francisco: Jossey-Bass.

[R38] Israel Barbara A., Schulz Amy J., Parker Edith A., Becker Adam B., and Community-Campus Partnerships for Health. 2001 Community-based participatory research: Policy recommendations for promoting a partnership approach in health research. Education for Health (Abingdon, England) 14: 182–97. 10.1080/13576280110051055 14742017

[R39] James Rosalina, Tsosie Rebecca, Sahota Puneet, Parker Myra, Dillard Denise, Sylvester Ileen, Lewis John, Klejka Joseph, Muzquiz LeeAnna, Olsen Polly, and 2014 Exploring pathways to trust: A tribal perspective on data sharing. Genetics in Medicine: O cial Journal of the American College of Medical Genetics 16: 820–26. 10.1038/gim.2014.47 PMC4224626 24830328

[R40] Johansson Patrick, Knox-Nicola Patricia, and Schmid Kendra. 2015 The Waponahki Tribal Health Assessment: Successfully Using CBPR to Conduct a Comprehensive and Baseline Health Assessment of Waponahki Tribal Members. Journal of Health Care for the Poor and Underserved 26: 889–907. 26320921 10.1353/hpu.2015.0099

[R41] Jumper-Reeves Leslie, Dustman Patricia A., Harthun Mary L., Kulis Stephen, and Brown Ed F.. 2014 American Indian cultures: How CBPR illuminated intertribal cultural elements fundamental to an adaptation effort. Prevention Science: The Offcial Journal of the Society for Prevention Research 15: 547–56. 10.1007/s11121-012-0361-7 PMC3726553 23412946

[R42] Kelley Allyson, Medicine Bull LaDawn Kay, and Lafranier Gary. 2016 Participatory visual methods for American Indian communities and mental health conversations. American Indian and Alaska Native Mental Health Research (Online) 23: 47–64. 28562842 10.5820/aian.2301.2016.47

[R43] Koenig Alexa, and Steinberg Jonathan. 2008 Federalism and the State Recognition of Native American Tribes: A Survey of State-Recognized Tribes and State Recognition Processes across the United States. Santa Clara Law Review 48: 79–153.

[R44] LaVeaux Deborah, and Christopher Suzanne. 2009 Contextualizing CBPR: Key Principles of CBPR meet the Indigenous research context. Pimatisiwin 7: 1. 20150951 PMC2818123

[R45] Legaspi Augusto, and Orr Eliza. 2007 Disseminating research on community health and well-being: A collaboration between Alaska Native villages and the academe. American Indian and Alaska Native Mental Health Research (Online) 14: 24–43. 17602411

[R46] Levac Danielle, Colquhoun Heather, and O’brien Kelly K.. 2010 Scoping studies: Advancing the methodology. Implementation Science: IS 5: 69. 20854677 10.1186/1748-5908-5-69 PMC2954944

[R47] Mariella Patricia, Brown Eddie, Carter Michael, and Verri Vanessa. 2009 Tribally-Driven Participatory Research: State of the practice and potential strategies for the future. Journal of Health Disparities Research and Practice 3: 4.

[R48] Matloub Jackie, Creswell Paul D., Strickland Rick, Pierce Kimmine, Stephenson Laura, Waukau Jerry, Kaur Judith S., and Remington Patrick. 2009 Lessons learned from a community-based participatory research project to improve American Indian cancer surveillance. Progress in Community Health Partnerships: Research, Education, and Action 3: 47–52. 10.1353/cpr.0.0058 20208301

[R49] Mello Michelle M., and Wolf Leslie E.. 2010 The Havasupai Indian tribe case—Lessons for research involving stored biologic samples. The New England Journal of Medicine 363: 204–7. 20538622 10.1056/NEJMp1005203

[R50] Morales Chelsea T., Muzquiz LeeAnna I., Howlett Kevin, Azure Bernie, Bodnar Brenda, Finley Vernon, Incashola Tony, Mathias Cheryl, Laukes Cindi, Beatty Patrick, and 2016 Partnership with the Confederated Salish and Kootenai Tribes: Establishing an Advisory Committee for Pharmacogenetic Research. Progress in Community Health Partnerships: Research, Education, and Action 10: 173–83. 10.1353/cpr.2016.0035 PMC5015644 27346763

[R51] Morton Deborah J., Proudfit Joely, Calac Daniel, Portillo Martina, Lofton-Fitzsimmons Geneva, Molina Theda, Flores Raymond, Lawson-Risso Barbara, and Majel-Mccauley Romelle. 2013 Creating research capacity through a tribally based institutional review board. American Journal of Public Health 103: 2160–64. 24134381 10.2105/AJPH.2013.301473 PMC3828979

[R52] Orians Carlyn E., Erb Julie, Kenyon Kathryn L., Lantz Paula M., Liebow Edward B., Joe Jennie R., and Burhansstipanov Linda. 2004 Public education strategies for delivering breast and cervical cancer screening in American Indian and Alaska Native populations. Journal of Public Health Management and Practice: JPHMP 10: 46–53. 15018341 10.1097/00124784-200401000-00009

[R53] Pacheco Christina M., Daley Sean M., Brown Travis, Filippi Melissa, Greiner Allen K., and Daley Christine M.. 2013 Moving Forward: Breaking the Cycle of Mistrust between American Indians and Researchers. American Journal of Public Health 103: 2152–59. 24134368 10.2105/AJPH.2013.301480 PMC3828980

[R54] Perry Cynthia, and Homan Barbara. 2010 Assessing tribal youth physical activity and programming using a community-based participatory research approach. Public Health Nursing (Boston, Mass.) 27: 104–14. 10.1111/j.1525-1446.2010.00833.x PMC2921582 20433664

[R55] Quigley Dianne, Sanchez Virginia, Handy D, Goble Robert, and George P. 2000 Participatory Research Strategies in Nuclear Risk Management for Native Communities. Journal of Health Communication 5: 305–31. 11191016 10.1080/10810730050199123

[R56] Quinn William W. 1990 Federal Acknowledgment of American Indian Tribes: The Historical Development of a Legal Concept. The American Journal of Legal History 34: 331–64.

[R57] Ravenscroft Julia, Schell Lawrence M., and Cole Tewentahawihothao. 2015 Applying the community partnership approach to human biology research. American Journal of Human Biology: The Offcial Journal of the Human Biology Council 27: 6–15. 10.1002/ajhb.22652 25380288

[R58] Rhoades Everett R., and Rhoades Dorothy A.. 2014 The public health foundation of health services for American Indians & Alaska Natives. American Journal of Public Health 104 S3: S278–85. 24758580 10.2105/AJPH.2013.301767 PMC4035891

[R59] Rink Elizabeth, Fourstar Kris, Medicine Elk Jarrett, Dick Rebecca, Jewett Lacey, and Gesink Dionne. 2012 Pregnancy prevention among American Indian men ages 18 to 24: The role of mental health and intention to use birth control. American Indian and Alaska Native Mental Health Research (Online) 19: 57–75. 22569725 10.5820/aian.1901.2012.57

[R60] Ross Lainie Friedman, Loup Allan, Nelson Robert M., Botkin Je rey R., Kost Rhonda, Smith George R., and Gehlert Sarah. 2010 Human Subjects Protections in Community-Engaged Research: A Research Ethics Framework. Journal of Empirical Research on Human Research Ethics 5: 5–17. 20235860 10.1525/jer.2010.5.1.5 PMC2946318

[R61] Salazar Martha. 2016 State Recognition of American Indian Tribes. National Conference of State Legislatures Legisbrief 24: 39.

[R62] Schell Lawrence M., Ravenscroft Julia, Cole Maxine, Jacobs Agnes, Newman Joan, and Akwesasne Task Force on the Environment. 2005 Health disparities and toxicant exposure of Akwesasne Mohawk young adults: A partnership approach to research. Environ Health Perspect 113: 1826–32. 16330372 10.1289/ehp.7914 PMC1314929

[R63] Schell Lawrence M., Ravenscroft Julia, Gallo Mia, and Denham Melinda. 2007 Advancing biocultural models by working with communities: A partnership approach. American Journal of Human Biology: The Offcial Journal of the Human Biology Council 19: 511–24. 10.1002/ajhb.20611 17546616

[R64] Schroepfer Tracy A., Matloub Jacqueline, Creswell Paul, Strickland Rick, and Anderson Diane M.. 2009 A community-specific approach to cancer research in Indian country. Progress in Community Health Partnerships: Research, Education, and Action 3: 317–25. 10.1353/cpr.0.0096 PMC3049894 20097993

[R65] Simonds Vanessa W., and Christopher Suzanne. 2013 Adapting Western research methods to indigenous ways of knowing. American Journal of Public Health 103: 2185–92. 23678897 10.2105/AJPH.2012.301157 PMC3828951

[R66] Thomas Lisa R., Donovan Dennis M., Sigo Robin L., Austin Lisette, and Marlatt Alan G.. 2009 The Community Pulling Together: A Tribal Community-University Partnership Project to Reduce Substance Abuse and Promote Good Health in a Reservation Tribal Community. Journal of Ethnicity in Substance Abuse 8: 283–300. 20157631 10.1080/15332640903110476 PMC2821063

[R67] Thomas Lisa Rey, Rosa Carmen, Forcehimes Alyssa, and Donovan Dennis M.. 2011 Research partnerships between academic institutions and American Indian and Alaska Native Tribes and organizations: Effective strategies and lessons learned in a multisite CTN study. The American Journal of Drug and Alcohol Abuse 37: 333–38. 21854275 10.3109/00952990.2011.596976 PMC3465683

[R68] Tingey Lauren, Cwik Mary F., Goklish Novalence, Larzelere-Hinton Francene, Lee Angelita, Suttle Rosemarie, Walkup John T., and Barlow Allison. 2014 Risk pathways for suicide among Native American adolescents.Qualitative Health Research 24: 1518–26. 25168705 10.1177/1049732314548688

[R69] United Nations General Assembly. 2007 United Nations Declaration on the Rights of Indigenous Peoples: Resolution/Adopted by the General Assembly, 13 September 2007. A/RES/61/295. Available online: https://www.un.org/development/desa/indigenouspeoples/declaration-on-the-rights-of-indigenous-peoples.html (accessed on 20 April 2019).

[R70] Urban Indian Health Commission. 2007 Invisible Tribes: Urban Indians. Seattle: Urban Indian Health Commission.

[R71] Urban Indian Health Commission. 2007 Invisible Tribes: Urban Indians and Their Health in a Changing World. Seattle: Urban Indian Health Commission.

[R72] US Department of Health and Human Services. 2018 US Department of Health and Human Services. 2018 Profile: American Indian/Alaska Native. Available online: https://www.minorityhealth.hhs.gov/omh/browse.aspx?lvl=3&lvlid=62 (accessed on 24 October 2018).

[R73] US Government Publishing Office. 2017 25 CFR 83 Procedures for Federal Acknowledgement of Indian Tribes. Available online: Tribes. Available online: https://www.govinfo.gov/app/details/CFR-/CFR-2017-title25-vol1/CFR-2017-title25-vol1-part83/context (accessed on 3 April 2019).

[R74] Valeggia Claudia R., and Josh Snodgrass J. 2015 Health of Indigenous Peoples. Annual Review of Anthropology 44: 117–35.

[R75] Walters Karina L., Lamarr June, Levy Rona L., Pearson Cynthia, Maresca Teresa, Mohammed Selina A., Fredriksen-Goldsen Karen, Sheryl Fryberg, and Simoni M, Evans-Campbell Teresa, Fredriksen-Goldsen Jane Karen, Fryberg Sheryl, and Jobe Jared B.. 2012 Project həli?dx w/Healthy Hearts Across Generations: Development and evaluation design of a tribally based cardiovascular disease prevention intervention for American Indian families. The Journal of Primary Prevention 33: 197–207. 22965622 10.1007/s10935-012-0274-z PMC3505854

[R76] Warne Donald K., and Frizzell Linda B.. 2014 American Indian health policy: Historical trends and contemporary issues. American Journal of Public Health 104 S3: S263–67. 10.2105/AJPH.2013.301682 PMC4035886 24754649

[R77] Wolf Leslie E., Walden Janice F., and Lo Bernard. 2005 Human subjects issues and IRB review in practice-based research. Annals of Family Medicine 3 S1: S30–37. 15928216 10.1370/afm.302 PMC1466958

[R78] Wolinetz Carrie D., and Collins Francis S.. 2017 Single-Minded Research Review: The Common Rule and Single IRB Policy. The American Journal of Bioethics: AJOB 17: 34–36. 10.1080/15265161.2017.1328542 PMC6688160 28661736

